# Effect of Post Treatment For Cu-Cr Source/Drain Electrodes on a-IGZO TFTs

**DOI:** 10.3390/ma9080623

**Published:** 2016-07-27

**Authors:** Shiben Hu, Zhiqiang Fang, Honglong Ning, Ruiqiang Tao, Xianzhe Liu, Yong Zeng, Rihui Yao, Fuxiang Huang, Zhengcao Li, Miao Xu, Lei Wang, Linfeng Lan, Junbiao Peng

**Affiliations:** 1 Institute of Polymer Optoelectronic Materials and Devices, State Key Laboratory of Luminescent Materials and Devices, Department of Materials Science and Engineering School, South China University of Technology, Guangzhou 510640, China; hushiben@foxmail.com (S.H.); fangzq1230@126.com (Z.F.); 564717456@qq.com (R.T.); 1057000149@qq.com (X.L.); 15360452751@163.com (Y.Z.); xumiao4049@126.com (M.X.); mslwang@scut.edu.cn (L.W.); lanlinfeng@scut.edu.cn (L.L.); psjbpeng@scut.edu.cn (J.P.); 2 School of Materials Science and Engineering, Chongqing University of Technology, Chongqing 400054, China; hfx@cqut.edu.cn; 3 State Key Laboratory of New Ceramic and Fine Processing, Tsinghua University, Beijing 100084, China; zcli@tsinghua.edu.cn

**Keywords:** interfaces, semiconductors, a-IGZO, Cu-Cr, TFTs, electrodes

## Abstract

We report a high-performance amorphous Indium-Gallium-Zinc-Oxide (a-IGZO) thin-film transistor (TFT) with new copper-chromium (Cu-Cr) alloy source/drain electrodes. The TFT shows a high mobility of 39.4 cm${}^{2}$·V${}^{-1}$·s${}^{-1}$ a turn-on voltage of −0.8 V and a low subthreshold swing of 0.47 V/decade. Cu diffusion is suppressed because pre-annealing can protect a-IGZO from damage during the electrode sputtering and reduce the copper diffusion paths by making film denser. Due to the interaction of Cr with a-IGZO, the carrier concentration of a-IGZO, which is responsible for high mobility, rises.

## 1. Introduction

The growing demands of high resolution (≥8 K), fast frame rate (≥480 Hz) and large panel size (≥110 inch) for flat panel displays need a thin-film transistor (TFT) array with high mobility and low resistance-capacitance (RC) delay [1,2]. Commercial display panels can utilize amorphous Indium-Gallium-Zinc-Oxide (a-IGZO) thin-film transistors (TFTs) to meet the high mobility requirement [3]. The replacement of conventional aluminum bus line by copper (Cu) bus line is urgent because the former’s low resistivity can adequately decrease RC delay to avoid image distortion and shading [4,5]. However, there are many reports that suggest that the inter-diffusion between Cu and a-IGZO deteriorate the electrical performance [6,7,8]. Meanwhile, pure Cu films exhibit poor adhesion to many substrates, such as glass and SiO${}_{2}$ [9,10,11]. Some papers use a barrier layer such as Ta, Ti, and Mo to prevent copper diffusing into IGZO and enhance the adhesion strength [12,13,14]. However, these technologies will increase manufacturing cost and difficulty of patterning because the etch rate of Cu and barrier layer metal (Ta, Ti, Mo, etc.) have an impact.

In this paper, we investigate the effect of copper-chromium (Cu-Cr) alloy source/drain electrodes (S/D). The introduction of Cr enhances the mobility to 39.4 cm${}^{2}$·V${}^{-1}$·s${}^{-1}$ which is 6 times higher than that of the TFT with no post treatment. The improvement can be attributed to the suppression of copper diffusion, and the interaction between Cr and IGZO. Meanwhile, Cu-Cr film exhibits good adhesion with glass.

## 2. Experiments

**Figure 1:**
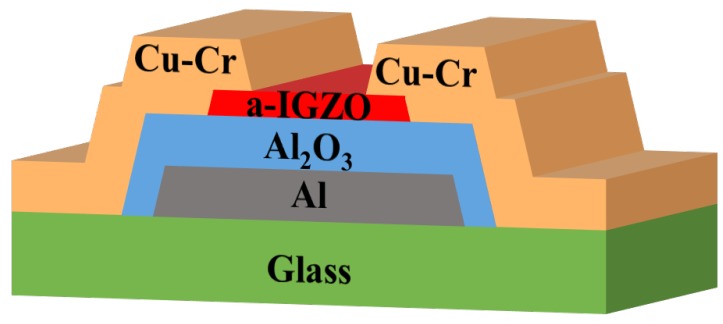
Schematic cross-sectional image of the amorphous Indium-Gallium-Zinc-Oxide (a-IGZO) thin-film transistors (TFTs).

The bottom-gate top-contact a-IGZO based TFTs were prepared on a glass substrate, as shown in Figure 1. A 200-nm-thick Al layer was deposited by DC magnetron sputtering as the gate electrode, then patterned by wet etching. Subsequently, a gate insulator layer of 200 nm-thick Al${}_{2}$O${}_{3}$ film formed on the surface of the Al by anodizing in an electrolyte consisting of 3.68 wt % ammonium tartrate solution and ethylene glycol [15]. Next, an 18 nm thick a-IGZO film was deposited by RF magnetron sputtering and patterned through the shadow mask. After the deposition, the devices were annealed at 400 ${}^{\circ }$C for 1 h in air. An alloy layer of 200 nm thick Cu-Cr film as source/drain (S/D) electrodes was prepared by DC magnetron sputtering using a Cu-Cr target (Cu-0.45 wt % Cr), and patterned through a shadow mask. Finally, the devices suffered a post annealing of 300 ${}^{\circ }$C for 1 h under argon (Ar) ambient.

## 3. Results and Discussion

**Figure 2:**
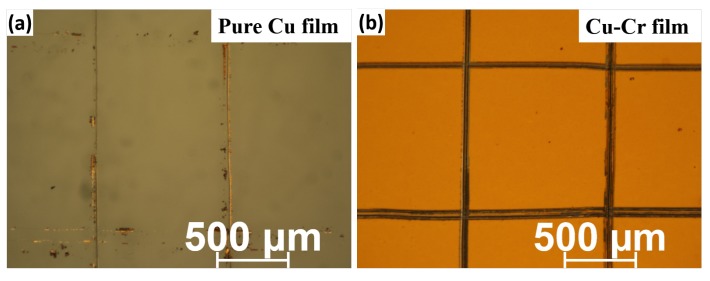
(**a**) Pure copper (Cu) and (**b**) copper-chromium (Cu-Cr) film image after tape test.

For evaluating the adhesion strength of Cu-Cr and pure Cu films on glass, a tape test is applied to these films [16]. The pure Cu samples are totally removed from the glass while the Cu-Cr films exhibit good adhesion which remains 100% on the glass, as shown in Figure 2. Because of the low solubility for Cr in Cu (0.8% at 1075 ${}^{\circ }$C), annealing can be expected to have Cr precipitates at the interface and form CrOx to enhance the adhesion.

**Figure 3:**
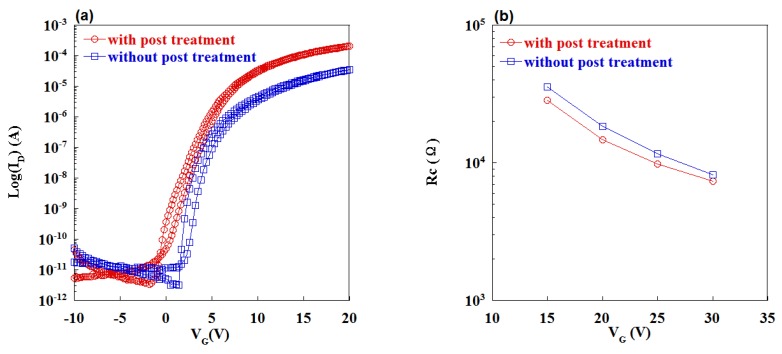
(**a**) Transfer curves and (**b**) variations of contact resistance for a-IGZO TFTs with Cu-Cr source/drain (S/D) electrodes.

The transfer curves of TFTs with Cu-Cr electrodes were measured under the drain voltage of 10.1 V, as presented in Figure 3a. The TFT parameters are extracted from it. The TFT with post treatment shows a high saturated mobility (*μ*${}_{sat}$) of 39.4 cm${}^{2}$·V${}^{-1}$·s${}^{-1}$, a subthreshold swing (SS) of 0.47 V/decade and a turn-on voltage (V${}_{on}$) of −0.8 V. Meanwhile, the TFT without post annealing shows a *μ*${}_{sat}$ of 6.4 cm${}^{2}$·V${}^{-1}$·s${}^{-1}$, an SS of 0.26 V/decade and a V${}_{on}$ of 1.4 V. The mobility of TFT with post annealing exhibits a significant improvement compared with the previous report which shows a mobility of 16 cm${}^{2}$·V${}^{-1}$·s${}^{-1}$ [4]. The contact resistance (Rc) of TFTs was extracted by transmission line method, as shown in Figure 3b. The Rc value of the Cu-Cr alloy electrodes device with post treatment is lower than that of the device without post treatment.Therefore, the higher mobility may result from the lower Rc.

**Figure 4:**
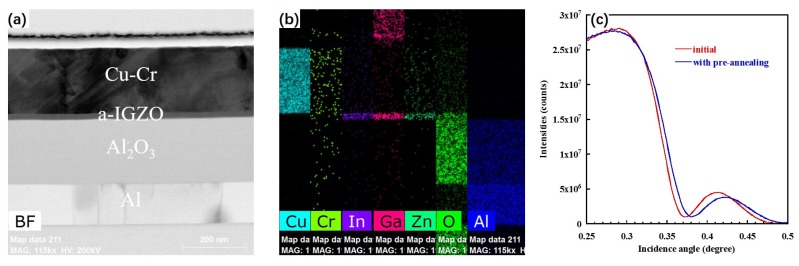
(**a**) Cross-sectional high resolution transmission electron microscope(HRTEM) image for a-IGZO TFT; (**b**) Cu, Cr, In, Ga, Zn, O and Al distribution detected by Energy-dispersive X-ray spectroscopy(EDS) mapping scan; (**c**) X-ray reflectivity(XRR) curves for a-IGZO film before and after suffering pre-annealing.

According to previous reports, Cu atoms diffused into the channel layer would degrade the TFT performance [6,7,8]. However, in our TFT, the device shows excellent performance. In order to calculate the improvement, a focused ion beam transmission electron microscope (FIB-TEM) is utilized to analyze the interface of Cu-Cr and IGZO. We can observe a smooth interface at Cu-Cr S/D and a-IGZO layer, which indicates that the deposition of Cu-Cr S/D did not damage the a-IGZO film, shown in Figure 4a. In Figure 4b, it is clear that the signal of Cu element is exactly limited on the S/D side, which indicates no diffusion of Cu. According to the X-ray reflectivity curves for the a-IGZO film before and after suffering pre-annealing shown in Figure 4c, it is clear that the critical angle of a-IGZO film with pre-annealing is larger than those that are as-deposited. This indicates that the pre-annealing process makes a-IGZO film denser than as-deposited film. Therefore, we can conclude that the prevention of Cu diffusion results from the pre-annealing process, which can efficiently protect the a-IGZO layer from damage during the subsequent deposition of the Cu-Cr electrode and decrease the diffusion paths of the Cu ion.

**Figure 5:**
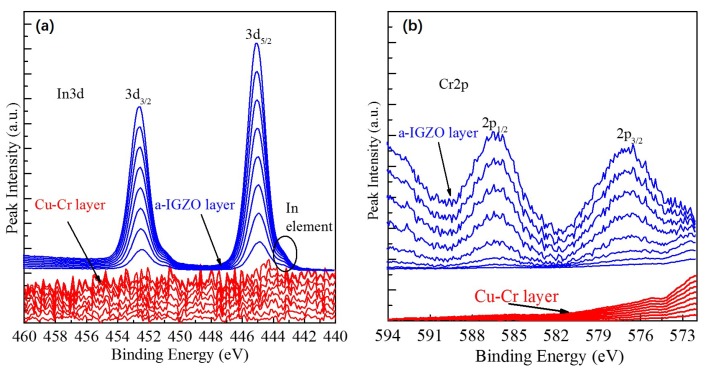
In3d (**a**) and Cr2p (**b**) level spectra of XPS depth profiles obtained from the Cu-Cr layer to a-IGZO layer regions.

To further elucidate the interaction between a-IGZO and Cu electrode doping into Cr, the XPS depth profile of the sample with Cu-Cr/a-IGZO structure was carried out. In the experiment, a sample of Cu-Cr (30 nm)/a-IGZO (18 nm) films was prepared on glass by DC and RF magnetron sputtering, respectively. Notably, the sample suffered the same annealing process which includes pre-annealing (400 ${}^{\circ }$C for 1 h in air) after the deposition of a-IGZO and post-annealing (300 ${}^{\circ }$C for 1 h in Ar ambient). Figure 5 shows core In3d and Cr2p level spectra of XPS depth profiles obtained from the Cu-Cr layer to a-IGZO layer regions. In the In3d spectra, a series of distinct peaks with lower binding energy are observed on the side of the a-IGZO layer corresponding to the free In element which indicates more oxygen vacancies. Meanwhile, the Cr2p peak can be observed only on the side of the a-IGZO layer, as shown in Figure 5b. The Cr2p${}_{1/2}$ energies (574 to 579 eV) could be ascribable to Cr oxide. These results reveal that Cr diffused into the a-IGZO film during the post-annealing phase. Then, oxygen was consumed from the a-IGZO which increased the concentration of oxygen vacancies and produced Cr oxide and a free In element. As we know, most of the carriers in the a-IGZO film are generated by oxygen vacancies. Thus, the high mobility of TFT can be attributed to the increase of carrier concentration in a-IGZO by the interaction between Cr and a-IGZO. This interpretation is consistent with the fact that the formation enthalpy of Cr${}_{2}$O${}_{3}$ (ΔH${}_{Cr_{2}O_{3}}$ = −1139.7 kJ/mol) is smaller than In${}_{2}$O${}_{3}$ (ΔH${}_{In_{2}O_{3}}$ = −925.8 kJ/mol), Ga${}_{2}$O${}_{3}$ (ΔH${}_{Ga_{2}O_{3}}$ = −1089 kJ/mol) and ZnO (ΔH${}_{ZnO}$ = −350.5 kJ/mol) [17]. The part-formation of Cr oxide acts as defect sites which lead to a larger SS.

## 4. Conclusions

In conclusion, we revealed the effect of Cr doping into Cu S/D electrodes on the performance of the TFT. The new Cu-Cr alloy contacted device shows a *μ*${}_{sat}$ of 39.4 cm${}^{2}$·V${}^{-1}$·s${}^{-1}$, an SS of 0.47 V/decade and a V${}_{on}$ of −0.8 V. Cu diffusion is suppressed because pre-annealing can protect a-IGZO from damage during the electrode sputtering and reduce the copper diffusion paths by making film denser. Due to the interaction of Cr to a-IGZO, the carrier concentration of a-IGZO rises, which enhances the electrical performance of TFT. Therefore, Cu-Cr Alloy S/D electrodes are a promising approach to achieve high-performance a-IGZO TFTs.
